# Fatigue-Related Factors for Community-Dwelling Older Adults with Diabetes: A Theory-Guided Multi-Dimensional Approach Using the Dynamic Biopsychosocial Model

**DOI:** 10.3390/ijerph16224502

**Published:** 2019-11-15

**Authors:** Hyerang Kim, Heesook Son

**Affiliations:** Red Cross College of Nursing, Chung-Ang University, Seoul 06974, Korea; hkim167@hotmail.com

**Keywords:** fatigue, older adults with diabetes, dynamic biopsychosocial model

## Abstract

Older adults with diabetes appear more susceptible to fatigue compared to younger adults with diabetes or healthy older adults, since aging and diabetes independently and synergistically influence fatigue. Few studies have investigated fatigue in older adults with diabetes using a multidimensional approach. This study explored the influences of physical, psychological, interpersonal, and contextual factors on diabetes fatigue using a dynamic biopsychosocial model. Face-to-face surveys were administered to community-dwelling older adults with diabetes and included variables across four domains (i.e., physical, psychological, interpersonal, and contextual factors). Univariate analyses and multiple linear regression were used. The mean fatigue score was 3.94 (standard deviation (SD) = 1.81) out of 7, and the prevalence of fatigue was 48.8%. Significant differences in fatigue severity by psychological, interpersonal, and contextual factors were found. Comorbidity and psychological factors were significant predictors of fatigue in the model, explaining 31.9% of the variance. As nearly half the sample experienced moderate or severe fatigue, which was significantly influenced by both comorbidity and psychological factors, including depression, sleep quality, and diet-related psychological characteristics, assessing patients’ psychological status may be important. Awareness of fatigue could be incorporated into dietary interventions for older adults with diabetes.

## 1. Introduction

South Korea is experiencing rapid growth in the aging population. In 2018, residents aged 65 years and older represented 14.3% of the total population, and the aging population will rise to be over 21% of the total population over the next decade [1]. The substantial aging population creates burdens for the management of chronic diseases for both individuals and society by increasing the risks for functional impairment and disabilities [2]. Among the chronic disease, diabetes is one of the most common in older adults, with approximately 18.8% of adults aged 65 years or older having diabetes worldwide, which is expected to double by 2045 [3]. In Korea, the prevalence of diabetes among older adults was reported to be 28.3% in 2018, which is expected to speed up with the rapid growth of the aging population [3,4].

Diabetes in older adults is unique and different from the presentation of diabetes in other age groups, as it is simultaneously influenced by both the degenerative aging process and abnormal glucose metabolism [5,6,7]. Both aging and diabetes independently increase an individual’s risk of impairment in their functional abilities in daily living and psychological well-being [7,8]. Having diabetes is a stressful life event that increases the burdens for older adults owing to the daily therapeutic regimen for diabetes and diminishes older adults’ functional abilities [9,10]. The double burden of aging and diabetes makes older adults more vulnerable physically, psychologically, and socially, and thus, reduces their perceived health status and quality of life [8].

Fatigue is a commonly experienced condition of daily life and has been defined in various ways depending on the context. It often refers to tiredness, lack of energy, or weariness [11,12]. Research suggests that approximately 20% of adults experience fatigue, which generally increases with age [13]. Furthermore, fatigue is a persistent complaint of diabetic individuals and is reported more than twice as much as in non-diabetic individuals [14,15]. Considering that aging and diabetes are independent risk factors for fatigue, older adults with diabetes may be more susceptible to fatigue, compared to both younger adults with diabetes and older adults without diabetes [8]. However, little data is available on the prevalence and severity of fatigue in older adults with diabetes.

Diabetes fatigue has vicious cyclic relationship with numerous factors, including diabetes symptoms, diabetic complications, other endocrine disorders, emotional distress, and lifestyle factors [16] and, in turn, it negatively influences emotions, lifestyle, and blood sugar control, increasing the risks for physical and psychological diabetes complications [17]. While diabetes fatigue has been conceptualized in several ways [16,17,18,19], the existing conceptual frameworks have been limited in their ability to comprehensively encompass the multidimensional characteristics of fatigue, including those related to biological, psychological, social, and environmental contexts. The dynamic biopsychosocial (DBPS) model provides a dynamic, ecological perspective of health, which views health as being determined by the interactions between biological, psychological, and social dynamics [20], and could be a useful conceptual framework for understanding the multidimensional properties of diabetes fatigue. Thus, the purpose of this study was to examine the multidimensional factors of fatigue for community-dwelling older adults with diabetes using the DBPS model.

### Theoretical Framework

The DBPS model is an expansion of the biopsychosocial model, which utilized an ecological perspective of the multidimensional health characteristics based on general systems theory [21,22]. Different from the biopsychosocial model, in the DBPS model, social factors were specifically divided into interpersonal factors and macrosystem contextual factors. The model explained human health as a consequence of the reciprocal influences of biological, psychological, interpersonal, and contextual factors. Each factor represents a set of interactive forces or systems that affect health. The impact of these factors on health is viewed as being dynamic, emphasizing that their levels of influence are continually changing over time rather than being fixed. The significance of the impact of each factor varies over time, which is referred to as centrality.

Biological dynamics capture the physical elements of the body that affect health. Each functional system is a complex, interconnected set of structures and cells that play a unique but reciprocal role in maintaining health. Psychological dynamics encompass multiple interdependent psychological factors, including variables of cognition, emotions, personality, attitudes, and behaviors that affect health. Interpersonal dynamics include the effects of actual and perceived social contacts on the dyadic and group processes affecting health. Interactive interpersonal dynamics include the entities with which an individual comes into direct contact (e.g., family members, work environments, peers, and community health resources) as well as the reverberating consequences of other’s actions that indirectly affect an individual’s health (e.g., spouses’ employment status and working environment and training of health care providers). Contextual dynamics include a broad pattern of shared culture, norms, policies, and values, which shape interpersonal, psychological, and biological factors.

**Figure 1:**
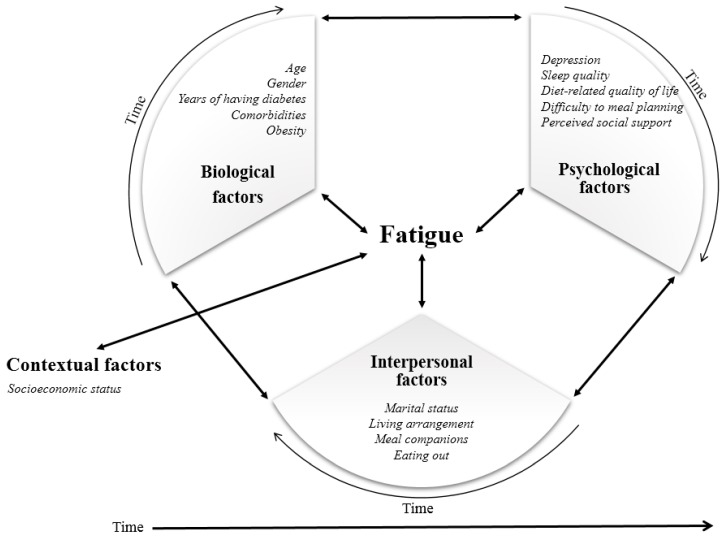
Conceptual framework of dynamic biopsychosocial determinants for fatigue.

In this study, diabetes- and aging-related biological, psychological, interpersonal, and contextual factors were incorporated in the DBPS model to investigate the multidimensional characteristics of fatigue in older adults with diabetes (Figure 1). The biological factors included age, gender, years with diabetes, having comorbidities, and obesity. Psychological factors included depression, sleep quality, diabetes diet-related quality of life, and perceived social support. Interpersonal factors included marital status, living arrangements, and having meal companions. Contextual factors included socioeconomic status, such as education level and household income.

## 2. Materials and Methods

### 2.1. Participants

Using a convenience sampling method, participants were recruited from a community health center and a senior center. Since the perception of fatigue may vary according to living situation owing to differences in clinical condition, functional independency of daily living practices and instrumental support [23,24], individuals who were living independently were included in our study. The inclusion criteria were: (1) having a diagnosis of diabetes and currently taking antihyperglycemic agents; (2) being aged 65 years or older; (3) having the cognitive ability to answer the survey questionnaires; and (4) living independently. The exclusion criteria were: (1) having a terminal disease or diagnosis with life expectancy less than six months; and (2) having been hospitalized or visited the emergency room at least twice due to acute hyperglycemic/hypoglycemic events or other acute inflammatory diseases in the three months prior to the study. After obtaining approval from the Institutional Research Board of the authors’ university (IRB No. 1041078-201901-HR-003-01), the face-to-face questionnaire surveys were conducted from March to May in 2019. Prior to the start of the study, participants were given the information about the study, including its purpose, procedure, the right to withdraw, and confidentiality, and the voluntary nature of their study participation. All study participants consented to participate.

The sample size was determined using G*Power 3.1.2 (Universität Düsseldorf, Germain) [25]. With a significance level and power set at 0.05 and 0.80, respectively, and an effect size at 0.15, the minimum sample size was estimated as 125. Estimating potential drop-out rate of 5%, the final sample size was determined to be 135. We collected 129 completed questionnaires and included 127 in the final statistical analysis after excluding 2 individuals with considerable missing responses.

### 2.2. Data Collection

The questionnaire consisted of four sections to measure each domain of the DBPS model. The first section included selected variables related to biological factors, such as age, gender, years with diabetes, the presence of comorbidities, and current height and weight. Obesity was determined using the estimation of body mass index (kg/m^2^), calculated with measured height (m) and weight (kg). In the second section, psychological characteristics such as depression, sleep quality, diabetes diet-related quality of life, and perceived social support were measured using validated instruments with good internal consistency and validity. The third section included items related to interpersonal factors, such as current marital status, living arrangement (“With whom do you live?”), presence of a meal companion (“With whom do you usually eat breakfast/lunch/dinner?”), and the frequency of eating out (times per week). The fourth section assessed contextual factors, including education level and household income.

### 2.3. Instruments

#### 2.3.1. Fatigue

Fatigue was assessed using the Fatigue Severity Scale (FSS), which was developed by Krupp and colleagues [26] and translated into Korean by Chung and Song [27]. The FSS was designed to measure fatigue distinguished from the characteristics of clinical depression and was known to be suitable for evaluating chronic functional fatigue by asking the effect of long-term accumulated fatigue on daily life. High reliability and validity were reported in previous studies [27,28]. It contained nine questions inquiring the extent of fatigue during the past week on a 7-point Likert-type scale (1 = not at all to 7 = extremely severe). The total mean score can be calculated by dividing the total sum of nine items by the number of items. A higher score indicates severe fatigue. The cut-off scores based on previous research were <4 points indicated normal levels of fatigue, 4–4.9 moderate fatigue, and ≥5 severe fatigue [14]. Cronbach’s alpha coefficients were 0.93 in the original study and 0.92 in this study.

#### 2.3.2. Depression

Depression was assessed using the Geriatric Depression Scale-15 (GDS-15), which was developed by Sheikh and Yesavage [29] and translated into Korean by Jang and colleagues [30]. It is a self-administered instrument that evaluates symptoms of depression and is designed especially for older adults. It has the advantage of having a relatively small number of items that are rated using the dichotomous responses of yes (1 point) or no (0 point), which has benefits considering attention and item comprehension [31]. The GDS-15 consists of 15 questions regarding how the respondent has felt over the past week. Out of a total score of 15 points, a score of 0–4 points was considered as non-depressed, 5–9 points as mild depression, and >9 points as severe depression [29]. Reliability estimated as KR-20 was *α* = 0.81 in this study.

#### 2.3.3. Sleep Quality

This was assessed using the Pittsburgh Sleep Quality Index (PSQI) developed by Buysse and colleagues [32]. It measures subjective sleep quality over the past month. It consists of 18 questions in seven areas, which includes overall subjective sleep quality, the duration for falling asleep, the actual sleep duration, the usual sleep efficiency, sleep disturbances, use of sleeping pills, and daytime dysfunction. Each area is scored according to the sleep-quality-index calculation method and was summed to evaluate the overall sleep quality. The higher the total score, the lower the sleep quality. A score of ≥5 points was used as the cut-off for having poor the sleep quality [32]. Cronbach’s alpha coefficient was 0.74 in this study.

#### 2.3.4. Diabetes Diet-Related Quality of Life

Diabetes diet-related psychological measure was assessed using Diabetes Diet-related Quality of Life–Revised version (DDRQOL-R-9), which was developed by Sato and colleagues [33]. The DDRQOL-R-9 consisted of nine items in three subscales: Satisfaction with Diet (three items), Burden of Diet Therapy (three items), and Perceived Merits of Diet Therapy (three items). The scale is rated on a 7-point Likert type scale ranging from 1 (very strongly disagree) to 7 (very strongly agree). The sum of the scores for each scale was evaluated in total scores of 100 points. The higher the score for each subscale, the better the Satisfaction with Diet and Perceived Merits of Diet Therapy, and the higher the Burden of Diet Therapy. DDRQOL-R-9 showed good internal consistency with Cronbach’s alpha coefficients of 0.86 for Satisfaction with Diet, 0.86 for Burden of Diet Therapy, and 0.82 for Perceived Merits of Diet Therapy in the original study. It was translated into Korean by a bilingual nursing researcher through a forward-translation and back-translation procedure. The internal consistency in this study was satisfactory (Cronbach’s alpha coefficient = 0.89 for Satisfaction with Diet, 0.87 for Burden of Diet Therapy, and 0.80 for Perceived Merits of Diet Therapy). A question inquiring about the difficulty planning meals was also asked to assess the psychological burden for engaging in daily dietary practices, using a 5-point Likert scale (1 = strongly disagree and 5 = strongly agree).

#### 2.3.5. Perceived Social Support

Perceived social support was assessed using the Multidimensional Scale of Perceived Social Support (MSPSS), which was developed by Zimet and colleagues [34]. This measures the extent to which an individual perceives social support from three different sources: family, friends, and significant others. It consists of 12 items rated on a 7-point Likert-type scale with scores ranging from 1 (very strongly disagree) to 7 (very strongly agree). Possible scores range from 4 to 28 on each of three subscales and from 12 to 84 for the total score, with higher scores representing higher perceived social support. The internal consistency and validity have been demonstrated in multiple study populations [34,35,36,37,38]. Cronbach’s alpha coefficient of the overall score in this study was 0.93, with subscales of 0.84, 0.92, 0.90 for family, friends, and significant others, respectively.

### 2.4. Data Analyses

The univariate analyses using one-way analyses of variance (ANOVAs) were conducted to examine the differences in fatigue by biological, psychological, social, and contextual profiles. Multiple linear regression analyses were conducted to determine if the variables in each domain predict fatigue. Using simultaneous multiple regression, all variables from each domain were included in a model to account for the potential confounding effect of other factors based on our proposed conceptual framework. The unstandardized B value was used as a measure of how strongly each predictor influences fatigue. A bootstrapping analysis in the regression models was conducted to estimate bias-corrected and accelerated (BCa) confidence intervals (CI) with 1000 replications. The values were used to determine the 95% CI for each variable. An effect was considered significant when the BCa CI did not include a zero. Adjusted *R*^2^ was used to assess the amount of variance in the domain score explained by the model. The variance inflation factors (VIF) was examined to evaluate the multicollinearity between the independent variables in the regression model. All statistical analyses were conducted using IBM SPSS Statistics version 25 (IBM Corp., Armonk, NY, USA). Statistical significance was set *p* < 0.05.

## 3. Results

Table 1 shows the severity and prevalence of fatigue for the participants. The mean score of fatigue was 3.94 (*SD* = 1.81, range 1–7). The prevalence of moderate and severe fatigue was 17.0% and 31.8%, respectively.

Based on the results of the univariate analysis using ANOVAs, differences in fatigue severity were found for the psychological, interpersonal, and contextual factors but not for biological factors (Appendix A). The severity of fatigue was significantly different between some psychological measures. The fatigue score was higher in those who were more depressive (*p* < 0.001), had poorer sleep quality (*p* = 0.001), and had difficulty in meal planning (*p* = 0.012). Among the subscales of diabetes diet-related quality of life, it was only found that the fatigue score was higher in those who were less satisfied with their diet (*p* = 0.004). There was no difference in fatigue by the median value of perceived social support. Among the variables characterized as interpersonal factors, there were no significant differences in fatigue by marital status and living arrangement, whereas significant differences by eating situation were found, like exclusively eating alone (*p* = 0.037) and frequency of eating out (*p* = 0.028). As to the contextual factors, those whose income was less than the minimum cost of living had higher levels of fatigue (*p* = 0.022).

Results of the multiple regression demonstrated that the significant predictors of fatigue severity were depression (*B* = 0.187, 95% CI (0.098, 0.276)), poor sleep quality (*B* = 0.642, 95% CI (−0.029, −1.255)), difficulty with meal planning (*B* = 0.233, 95% CI (0.051, 0.415)), and satisfaction with diet (*B* = −0.090, 95% CI (−0.172, −0.007); adjusted *R*^2^ = 0.290, *p* < 0.001; Table 2). The prediction models of biological, interpersonal, and contextual factors were not statistically significant.

Using simultaneous multiple regression, the final model included all variables to simultaneously adjust for biological, interpersonal, and contextual factors as potential confounding factors (Table 3). The results indicated that poor sleep quality (*B* = 0.762, 95% CI (0.095, 1.428)) was the most strongly associated with fatigue, followed by comorbidity (*B* = 0.752, 95% CI (0.096, 1.408)) (Table 3). Other psychological variables, including depression (*B* = 0.166, 95% CI (0.066, 0.265)), difficulty with meal planning (*B* = 0.291, 95% CI (0.091, 0.490)) and satisfaction with diet (*B* = −0.133, 95% CI (−0.219, −0.047)), remained significant in the model (Adjusted *R*^2^ = 0.319, *p* < 0.001). The influence of diet-related variables on fatigue increased after controlling for potential confounding factors.

## 4. Discussion

This study was guided by the DBPS model and examined the multidimensional factors that were related to fatigue in a sample of community-dwelling older adults with diabetes. The differences in the severity of fatigue were associated with psychological, interpersonal, and contextual factors, but not for biological factors. In the simultaneous multiple regression model, comorbidity and psychological factors, including depression, poor sleep quality, satisfaction with diet, and difficulties with meal planning, were identified as being significant predictors for fatigue severity in older adults with diabetes.

The prevalence and the severity of fatigue in this study were found to be higher than previous reports of fatigue in the general population of older adults [38,39]. Although it is generally acknowledged that aging itself is a risk factor for fatigue [11] and that older adults with diabetes are more vulnerable to fatigue compared to adults without diabetes [24,40], results have been inconclusive regarding the association between fatigue and aging [14,40,41]. Studies that have used the same instruments used in the current study showed mixed results regarding differences in the prevalence of fatigue between younger and older adults [14,41]. This study also did not find the presence of a significant relationship between fatigue and age in this sample of older adults, identifying no differences between the individuals aged 65–74 years and those aged 75 years or older. One possible explanation for the mixed results for the association of fatigue and age might be variations in the prevalence of self-reported fatigue across studies depending on the variance of the measurement tools, time frame for measurement, and characteristics of the study population (e.g., clinical or subclinical conditions, physical and psychological functionality, and sociocultural background) [40,42,43]. Another possible explanation could be that increased fatigue may be associated with a variety of age-related changes rather than being solely influenced by age itself [11]. For example, it is well acknowledged that diabetes, especially in older adults, is related to high comorbidity burden that increases the impairment of physical and psychosocial functionalities [5]. Thus, older adults with comorbidities are likely to be at increased risk of experiencing fatigue comorbidities [44]. In this study, more than 65% of participants had at least two comorbid chronic diseases, such as hypertension, arthritis, and dyslipidemia, and comorbidity was found to be the strongest predictor for fatigue among the predictors included in the model.

On the other hand, prevalence findings in this study were consistent with rates identified among the healthy older adults living independently without any chronic conditions that may be related to fatigue [39]. These similarities could be owing to having functional independence in the daily activities for living in our sample, as this study did not limit participants’ abilities to the independent daily activities for self-care and social activities. For this reason, the results suggest that functional status or performance may be more critical factors for fatigue rather than chronological age and disease itself [39,40,45].

In this study, both the prevalence of depression and fatigue among participants was 49%, which was higher than reported in a sample of healthy older adults in a previous study (depression in 44% and fatigue in 35%) [46]. In a study by Jain et al. [16], the prevalence of depression (53%) and fatigue (68%) in individuals with diabetes were significantly higher than in individuals without diabetes (19% for depression and 17% for fatigue). It has been found that older adults with diabetes are at increased risk for the comorbid diagnoses of depression and fatigue [18,47,48] with the risk of fatigue in older adults with depression being twice as high as those without depression [14]. However, the risk of having comorbid depression and fatigue increased threefold in older adults with diabetes [13]. The high comorbidity for fatigue and depression can be explained by their commonly shared risk factors [49]. Although there were differences in the variables examined depending on the scope and the purpose of the study, numerous factors have been identified as common factors for both fatigue and depression. For example, depressive older adults with diabetes commonly experience poor sleep quality [50], which may lead to daytime sleep or inadequate dietary intake and increase fatigue [51]. Furthermore, they have poor appetites and undesirable dietary patterns, leading to frequent meal skipping and inadequate and/or imbalanced dietary intake (e.g., high-carbohydrates, high-fat, and high-caffeine diet), which were known to be fatigue-induced diet [52,53]. In addition, economic strain or hardship, lack of social support, and social isolation are well-known risk factors for both depression and fatigue [39,54,55].

We found poor sleep quality was the second strongest predictor of fatigue in this sample of older adults with diabetes. Poor sleep quality and reduced sleep duration are well-known predictors of fatigue [56]. Deteriorated sleep quality is the most manifested aging-related characteristics, leading to negative consequences for health and quality of life [57]. Diabetes and poor sleep quality have a reciprocal relationship, indicating that poor sleep quality adversely influences insulin sensitivity and blood glucose control, while diabetic symptoms, such as nocturia, nocturnal hypoglycemia, and restless legs syndrome, causes sleep deprivation and fragmentation [58,59]. Research has reported that 40%–70% of the older population experienced sleep disorder or poor sleep quality [60]. The prevalence of sleep disorder was 1.4 times higher in diabetes patients, compared to those without diabetes [61]. In this study, 71.3% of the participants reported poor sleep quality with extended sleep latency and sleep fragmentation, which was higher than rates reported in younger adults with diabetes [62,63]. This finding indicates that older adults with diabetes are susceptible to poor sleep quality, which may have a negative association with fatigue. Given that poor sleep quality is associated with other psychological outcomes such as depression and poor quality of life as well as low adherence to diabetes self-management and diabetes outcomes [62], health care providers should pay increased attention to providing appropriate interventions to improve sleep quality in older adults with diabetes.

Satisfaction with diet and difficulty with meal planning, as the diet-related psychological factors, showed strong relationships with fatigue. Dietary factors, including healthy eating, dietary patterns, and eating behaviors, have been highlighted in the diabetes literature [64,65,66]. A recent study found that unhealthy diets led to excessive dietary energy intake, extreme dietary energy restriction, protein malnutrition, and starvation ketosis, and all of these are causes of diabetes fatigue [16]. Zhu and colleagues [67] reported the causal relationship of emotional eating with fatigue in adults with type 2 diabetes, but further investigation on how psychological aspects of dietary management are associated with diabetes fatigue is needed.

Satisfaction with diet is an essential component of older adults’ daily lives, especially their diet-related quality of life [68]. Satisfaction with one’s diet generally decreased with age [69] since degenerative aging-related physical and psychological changes negatively impact appetite and palatability as well as the physical ability to prepare food [70,71,72]. Previous research has shown lower satisfaction with diet in older adults with diabetes compared to younger adults aged 19–64 years using the same instrument [33]. An unacceptable or unpalatable diet can lead to poor dietary intake, resulting in malnutrition and can ultimately have adverse health effects [8]. Furthermore, individuals with diabetes are recommended to follow diabetes dietary guidelines that include controlled energy intake, carbohydrate counting, and a balanced diet [73,74]. Such changes may lead to the restriction of food choices and recipe options, which individuals have habitually, which may affect joy and satisfaction regarding one’s diet [73,75]. Dietary modifications and restrictions may be more challenging for older adults who have long-established food preferences and dietary habits, further decreasing their satisfaction with their diet [68] and is one of the reasons for the rejection or failure to adherence to diabetes dietary guidelines among older adults [76].

On the other hand, dietary management for diabetes is known to be the most problematic aspect of the disease, requiring daily engagement in dietary practice [77,78]. In fact, most of the individuals with diabetes were burdened by engaging in dietary self-management compared to engaging in other types of diabetes self-management like exercise, insulin injection, self-monitoring of blood sugar, and taking oral antihypertensive agents [79]. Thus, the burdens experienced as a result of dietary self-management on daily practice may vary but generally include challenges with dietary restriction, difficulties with meal planning, barriers to dietary practice, and failure to utilize dietary self-management [80].

The difficulties with meal planning were found to be another independent predictor for fatigue in this study. Older adults have more barriers to practice daily dietary self-management. The burden of economic hardship and poor physical functioning were reported as one major factor affecting daily dietary routines [76,81,82]. For example, older adults with diabetes usually experience a financial burden to buy high-quality foods for a balanced diet because their income is reduced after retirement [83]. Both declines in cognitive functioning and memory, as well as disabilities or impairments in physical functioning, may limit the ability to complete daily dietary routines, like food shopping, preparation, and cooking [52,55,84]. Furthermore, the lack of social support due to changes in family structure (e.g., loss of spouse and independence of their children), may contribute to an increased need for help and support with meal preparation in older adults [71]. These factors may lead to poor dietary adherence, which is associated with negative emotions, such as guilt and helplessness regarding dietary management [7]. Taken together, it is plausible that prolonged psychological burdens in relation to dissatisfaction with diet and difficulty with meal planning can influence the maladaptation of a diabetes diet regimen and exacerbate the motivation and attitude to dietary self-management as well as actual engagement in dietary self-management practices [85]. These factors may result in chronic emotional exhaustion, a type of fatigue [86,87].

The significance of this study can be demonstrated in terms of both its practical implications and research. First, this study highlighted the susceptibility to fatigue in older adults with diabetes, suggesting the need to provide specific care for fatigue management. Second, the DBPS model provided an integrated perspective of the psychological and social manifestations of fatigue that could not be explained by conventional biomedical perspectives. Based on an understanding of multiple dimensions of fatigue, this study suggested the need for a multidimensional approach to fatigue assessment and the development of fatigue intervention. This approach to conceptualizing fatigue could be applicable to understanding disease-related fatigue in other chronic diseases and different age groups. Third, this study was the first to describe the relationship between diet-related psychological factors and fatigue in diabetes, which has been insufficiently addressed in previous empirical research. The inclusion of psychological components in dietary intervention for improving diet-related psychological difficulties and satisfaction is needed rather than solely focusing on dietary intake and diet therapy itself.

However, the study has some limitations worth noting. Due to the limited number of participants, only the variables that were identified as being either diabetes- and aging-related factors for fatigue were included in the analysis. Furthermore, multiple studies with large samples are required to confirm our results and to identify other factors related to fatigue in this population. For example, variables, such as diet-related distress or stress, adherence to a dietary regimen, and actual dietary patterns and food consumption, are needed to comprehensively explain the mechanisms of how diet-related psychological factors affect fatigue. Another limitation regarding the study’s participants was that the sample was, in general, among healthy older adults who were actively engaged in social activities in their local health and senior centers. Further research should include older adults with different spectrums of biological, psychological, interpersonal, and contextual characteristics. Furthermore, this study only included older adults; thus, the comparison of the prevalence and the severity of fatigue across age groups was not available, and the differences in the factors by age groups could not be explored. We recommend a large-scale investigation, including different age groups, to provide a comprehensive understanding of age-specific factors of diabetes fatigue, which would contribute to the development of a tailored and effective diabetes fatigue intervention.

## 5. Conclusions

Using the DBPS model, the current study examined the influences of physical, psychological, interpersonal, and contextual factors on fatigue in a sample of community-dwelling older adults with diabetes. We found that almost half of the older adults with diabetes experienced moderate or severe fatigue, indicating susceptibility to fatigue in this population. In addition, comorbidity, psychological factors, particularly sleep quality and diet-related psychological variables, were strongly related to fatigue compared to the other factors. These results also highlighted the need for diet-specific psychological measures for fatigue, including dietary satisfaction or difficulties in dietary practice, and suggest that psychological components should be incorporated in dietary intervention for older adults with diabetes. These components might be helpful to support daily dietary practices as well as improve diet-related psychological burden and fatigue. Future research should examine the relationship between diet-specific psychological factors and fatigue across contexts to further explain the interactive, complex influence of multiple factors on fatigue.

## Figures and Tables

**Table 1:** Severity and prevalence of fatigue in older adults with diabetes (*N* = 127).

Level of Fatigue	Mean (*SD*; Range)	Frequency (%)
Fatigue (total score of 7 points)	3.94 (1.81; 1–7)	
Normal (<4)		66 (51.2)
Moderate fatigue (4–4.9)		22 (17.0)
Severe fatigue (≥5)		41 (31.8)

**Table 2:** Multiple regression analysis for predicting fatigue severity on each domain of the dynamic biopsychosocial (DBPS) model.

Predictors	Unstandardized		Standardized		Correlations		VIF
	*B* (95% CI)	*SE*	*β*	*p*	Partial	Part	
Biological							
Age	0.029 (−0.028, 0.086)	0.029	0.102	0.317	0.091	0.090	1.288
Gender	0.083 (−0.582, 0.748)	0.336	0.023	0.805	0.022	0.022	1.042
Years with diabetes	0.007 (−0.025,0.038)	0.016	0.042	0.672	0.038	0.038	1.231
Comorbidities	−0.090 (−0.365, 0.185)	0.139	−0.060	0.519	−0.058	−0.058	1.066
Body mass index	0.033 (−0.075, 0.141)	0.054	0.056	0.548	0.055	0.054	1.084
Model fit	Adjusted *R*^2^ = −0.024, *F* = 0.415, *p* = 0.838; Durbin-Watson: 0.588						
Psychological							
Depression	0.187 (0.098, 0.276)	0.045	0.368	<0.001	0.356	0.312	1.387
Poor sleep quality	0.642 (0.029, 1.255)	0.309	0.163	0.040	0.187	0.156	1.091
Difficulty with meal planning	0.233 (0.051, 0.415)	0.092	0.204	0.013	0.226	0.190	1.146
Satisfaction with diet	−0.090 (−0.172, −0.007)	0.042	−0.173	0.033	−0.194	−0.162	1.137
Burden of diet therapy	0.015 (−0.061, 0.092)	0.039	0.031	0.695	0.036	0.029	1.119
Perceived merits of diet therapy	0.066 (−0.016, 0.149)	0.042	0.128	0.115	0.144	0.119	1.156
Perceived social support	−0.001 (−0.014, 0.013)	0.007	−0.008	0.923	−0.007	−0.007	1.156
Model fit	Adjusted *R*^2^ = 0.290, *F* = 8.348, *p* < 0.001; Durbin-Watson: 1.026						
Interpersonal							
Marital status	0.174 (−0.779, 1.126)	0.481	0.048	0.719	0.032	0.032	2.323
Living arrangement	−0.075 (−1.132, 0.982)	0.534	−0.020	0.888	−0.013	−0.012	2.524
Exclusive eating alone	0.795 (−0.076, 1.666)	0.440	0.199	0.073	0.160	0.158	1.596
Eating out	0.137 (0.415, 0.144)	0.140	0.090	0.330	0.087	0.085	1.114
Model fit	Adjusted *R*^2^ = 0.024, *F* = 1.771, *p* = 0.139; Durbin-Watson: 0.583						
Contextual							
Education	−0.069 (−0.344, 0.205)	0.139	−0.050	0.617	−0.046	−0.046	1.158
Household income	−0.001 (−0.004, 0.001)	0.001	−0.118	0.234	−0.110	−0.110	1.158
Model fit	Adjusted *R*^2^ = 0.024, *F* = 1.771, *p* = 0.139; Durbin-Watson: 0.583						

**Table 3:** Simultaneous multiple regression of selected predictors for fatigue severity after adjusting by biological, interpersonal, and contextual factors.

Predictors	Unstandardized		Standardized		Correlations		VIF
	B (95 % CI)	SE	*β*	*p*	Partial	Part	
Comorbidity	0.752 (0.096, 1.408)	0.331	0.200	0.025	0.221	0.174	1.319
Depression	0.166 (0.066, 0.265)	0.050	0.329	0.001	0.312	0.253	1.685
Poor sleep quality	0.762 (0.095, 1.428)	0.336	0.191	0.026	0.220	0.174	1.211
Difficulty with meal planning	0.291 (0.091, 0.490)	0.101	0.254	0.005	0.276	0.221	1.315
Satisfaction with diet	−0.133 (−0.219, −0.047)	0.043	−0.261	0.003	−0.293	0.236	1.226
Model fit	Adjusted *R*^2^ = 0.319, *F* = 4.619, *p* < 0.001; Durbin-Watson: 1.142						

Variables for adjustment included age, gender, duration with diabetes, and comorbidity from biological domain, burden of diet therapy, perceive merits of diet therapy, and social support from psychological domain, living alone, eating alone, and eating out from interpersonal domain, and education and household income levels from contextual factors.

## Appendix A

**Table A1:** The severity of fatigue according to four domains of the DBPS model.

DBPS Domain	Potential Predictor			Fatigue	
			*n* (%)	*M (SD)*	*F(p)*
Biological	Age	<75-years-old	45 (35.4)	3.73 (1.79)	0.634 (0.428)
		≥75-years-old	82 (64.6)	4.00 (1.81)	
	Gender	Men	50 (39.4)	3.88 (1.57)	0.006 (0.937)
		Women	77 (60.6)	3.91 (1.94)	
	Years with diabetes	<10 years	58 (45.7)	3.82 (1.77)	0.192 (0.662)
		≥10 years	69 (54.3)	3.97 (1.84)	
	Comorbidities	<2	44 (34.6)	3.98 (1.83)	0.421 (0.518)
		≥2	83 (65.4)	3.76 (1.75)	
	Body mass index (kg/m^2^)	Normal (<23.0)	46 (36.2)	3.83 (1.88)	0.012 (0.988)
		Overweight (23.0–24.9)	34 (26.8)	3.89 (1.83)	
		Obesity (≥25.0)	44 (34.6)	3.88 (1.73)	
Psychological	Depression	Non-Depressed	63 (49.6)	3.30 (1.68)	12.475 (<0.001)
		Moderate Depression	45 (35.4)	4.11 (1.76)	
		Severe Depression	19 (15.0)	5.41 (1.24)	
	Sleep quality	Good	37 (29.1)	3.09 (1.53)	11.547 (0.001)
		Poor	90 (70.0)	4.24 (1.80)	
	Difficulty with meal planning	Not at all difficult	48 (37.8)	3.45 (1.83)	4.548 (0.012)
		Not so difficult/ Somewhat	39 (30.7)	3.78 (1.59)	
		Very difficult/ Extremely difficult	40 (31.5)	4.56 (1.80)	
	Satisfaction with diet	<median	68 (53.5)	4.32 (1.74)	8.509 (0.004)
		≥median	59 (46.5)	3.41 (1.75)	
	Burden of diet therapy	<median	65 (51.2)	4.00 (1.74)	0.407 (0.525)
		≥median	62 (48.8)	3.80 (1.87)	
	Perceived merits of diet therapy	<median	70 (55.1)	4.03 (1.81)	0.752 (0.387)
		≥median	57 (44.9)	3.75 (1.79)	
	Perceived social support	<median	60 (47.2)	4.12 (1.80)	1.739 (0.190)
		≥median	67 (52.8)	3.70 (1.79)	
Interpersonal	Marital status	Widowed/divorced/ separated	58 (45.7)	3.97 (1.84)	0.165 (0.685)
		Married/partnered	69 (54.3)	3.84 (1.78)	
	Living arrangement	Living alone	41 (32.3)	4.14 (1.88)	1.108 (0.295)
		Living with others	86 (67.7)	3.78 (1.76)	
	Exclusive eating alone *	No	92 (72.4)	3.70 (1.73)	4.431 (0.037)
		Yes	35 (27.6)	4.44 (1.89)	
	Eating out	Less than 2 times a week	93 (73.2)	4.11 (1.81)	4.972 (0.028)
		3 times or more a week	34 (26.8)	3.32 (1.66)	
Contextual	Education	<High School	64 (50.4)	4.01 (2.15)	0.512 (0.476)
		≥High School	63 (49.6)	3.78 (1.52)	
	Household income	≤1,000,000 Korean Won	73 (57.5)	4.21 (1.81)	5.365 (0.022)
		>1,000,000 Korean Won	54 (42.5)	3.48 (1.72)	

Note: *M*, mean; *SD*, standard deviation; Korean Won (1000 KW ≈ 1.2 USD); * Eating every meal alone.
